# No evidence of association between habitual physical activity and ECG traits: Insights from the electronic Framingham Heart Study

**DOI:** 10.1016/j.cvdhj.2021.11.004

**Published:** 2021-11-23

**Authors:** Jelena Kornej, Joanne M. Murabito, Yuankai Zhang, Chunyu Liu, Ludovic Trinquart, Mayank Sardana, Emily S. Manders, Michael M. Hammond, Nicole L. Spartano, Chathurangi H. Pathiravasan, Xuzhi Wang, Belinda Borrelli, David D. McManus, Emelia J. Benjamin, Honghuang Lin

**Affiliations:** ∗ National Heart, Lung, and Blood Institute and Boston University's Framingham Heart Study, Framingham, Massachusetts; † Section of Cardiovascular Medicine, Department of Medicine, Boston Medical Center, Boston University School of Medicine, Boston, Massachusetts; ‡ Section of General Internal Medicine, Department of Medicine, Boston University School of Medicine, Boston, Massachusetts; § Department of Biostatistics, Boston University School of Public Health, Boston, Massachusetts; ‖ Cardiology Division, Department of Medicine, University of California San Francisco, San Francisco, California; ¶ Section of Endocrinology, Diabetes, Nutrition, and Weight Management, Boston University School of Medicine, Boston, Massachusetts; # Henry M. Goldman School of Dental Medicine, Center for Behavioral Science Research, Department of Health Policy & Health Services Research, Boston University, Boston, Massachusetts; ∗∗ Cardiology Division, Department of Medicine, University of Massachusetts Medical School, Worcester, Massachusetts; †† Department of Quantitative Health Sciences, University of Massachusetts Medical School, Worcester, Massachusetts; ‡‡ Department of Epidemiology, Boston University School of Public Health, Boston, Massachusetts; §§ Section of Computational Biomedicine, Department of Medicine, Boston University School of Medicine, Boston, Massachusetts

**Keywords:** Epidemiology, Mobile health, Smartwatch, Lifestyle

**Key Findings**

• In the Framingham Heart Study, habitual physical activity measured as daily steps using Apple Watch is not associated with electrocardiogram traits PR interval, QRS interval, and QTc interval.

Physical activity (PA) improves risk factors associated with cardiovascular disease (high blood pressure, diabetes, obesity) and reduces the risk of heart failure, stroke, and coronary heart disease.^1^ Smartwatches are useful tools for monitoring habitual PA and may enhance phenotyping of community-dwelling individuals. Daily step count reported by smartwatches offers a helpful measure of overall PA capturing a variety of exercise types.

Electrocardiogram (ECG) traits are established cardiovascular risk markers, but limited knowledge is available regarding the association of habitual PA with ECG traits. ECG traits such as PR interval, QRS, and QT interval can be used to diagnose conduction disturbances and to predict cardiovascular diseases including sudden cardiac death, myocardial infarction, heart failure, and atrial fibrillation. Low PA is known to associate with poor cardiovascular health. We therefore hypothesized that ECG traits might act as an intermediate phenotype between PA and cardiovascular health. Specifically, we hypothesized that higher mean PR interval, QRS, and QTc interval would be associated with lower PA. The objective of the current analysis was to examine associations between average daily steps from the smartwatch and ECG traits among the electronic Framingham Heart Study (eFHS) participants.

The Framingham Heart Study (FHS) is an observational multigenerational cohort located in Framingham, Massachusetts. The eFHS cohort started enrolling participants from the Third Generation Cohort, multiethnic Omni 2, and New Offspring Spouse Cohort in June 2016 during their third research center examination.^2^ The Apple Watch was provided to participants as part of the eFHS if they owned an iPhone with a compatible iOS (version 9 or higher). Participants were also allowed to wear their own Apple Watch. Up to January 31, 2019, 1948 participants were enrolled with at least 12-month follow-up period. All participants underwent an ECG as a routine part of their examination. Clinical variables were also measured during the examination at the FHS Research Center. Habitual PA was measured as the average daily step count transmitted by the smartwatch (Apple Watch, Series 0) over a minimum of 30 days with at least 5 hours of wear time with steps. Participants with less than 30 active days were excluded (n = 186).

The association of average daily step with each ECG trait was estimated using linear regression models adjusting for age, sex, and wear time. In a secondary analysis, we further adjusted associations for clinical risk factors, including systolic and diastolic blood pressure, body mass index, current smoking, alcohol consumption, diabetes, antihypertensive treatment, prevalent atrial fibrillation, heart failure, and myocardial infarction. Significant associations were defined as those with 2-sided *P* value <.05/3 = .017 (3 = number of ECG traits tested).

The current study included 935 participants with available step and ECG data (mean age 53 ± 9 years, 60% women, mean body mass index 28.3 ± 5.5 kg/m^2^). The participants took a median of 7235 (25th percentile 5559; 75th percentile 9048) daily steps during a median study period of 325 days (Q1–Q3 136–527 days). The average time interval between ECG examination and the first day of PA measurement was 74 days. We examined the association of daily steps with 3 ECG traits, including PR interval (median 160 ms, interquartile range [IQR]: 146–178 ms), QRS (median 88 ms, IQR 82–94 ms), QTc (median 419 ms, IQR 406–432 ms).

As shown in Table 1, we did not find evidence of an association between any ECG trait with daily steps in either the primary or secondary models. We did not observe an age interaction between step count and ECG traits.

**Table 1:** Association between physical activity and electrocardiogram traits

ECG trait	Primary			Secondary		
	β†	95% CI	*P* value	β†	95% CI	P-value
PR interval, ms	-0.20	-0.82; 0.43	.53	0.16	-0.50; 0.82	0.63
QRS interval, ms	0.16	-0.14; 0.46	.29	0.26	-0.05; 0.57	0.11
QTc interval, ms	0.01	-0.49; 0.51	.96	0.18	-0.34; 0.70	0.50

Primary model: Adjusted for age, sex, and wearing time.

Secondary model: Additionally adjusted for diastolic and systolic blood pressure, body mass index, current smoking, alcohol consumption (>7/14 drinks weekly for females/males), diabetes, antihypertensive treatment, history of atrial fibrillation, heart failure, and myocardial infarction.

CI = confidence interval; ECG = electrocardiogram.

† β represents the change in ECG traits (ms) for every 1000 increase in daily steps.

The main finding of our analysis was that habitual PA measured as daily steps was not associated with ECG traits—PR interval, QRS interval, and QTc interval. Although previous research reported significantly shorter PR interval duration in aerobically nonfit compared to fit individuals, we did not find a significant association between habitual PA and PR interval in the current study. QRS duration is significantly associated with cardiovascular mortality, especially in individuals with complete bundle brunch block. Previous FHS studies reported that incomplete and complete bundle branch block were associated with up to 2-fold risk for heart failure, 4-fold risk for pacemaker implantation, and ischemia-induced ventricular tachycardia or fibrillation.^3^ Finally, QT prolongation could be considered as a surrogate parameter of subclinical atherosclerosis and can be predictive of future atherosclerotic vascular events, including stroke.^4^ Also, a previous study reported association between inactivity and low activity showing QTc interval prolongation in an older population.^5^

There are several limitations that we would like to acknowledge, which may explain the lack of association and why we did not confirm our hypothesis. Our study was cross-sectional, and our sample size was modest; we may have lacked power to detect a small effect. Habitual PA was not contemporaneous with the research center ECG; 72% of participants started to return PA data within 1 week, whereas the remaining participants started later. We also only assessed the total volume of PA (in steps/day) and did not examine whether the intensity of PA or other types of PA were related to ECG traits. In addition, eFHS participants are generally healthier and have lower cardiovascular comorbidity than the rest of FHS participants. The study population was of middle-aged participants mostly of European ancestry, limiting generalizability of our findings in other races/ethnicities. Finally, we acknowledge our hypothesis may be false.

In conclusion, despite some prior studies associating PA and ECG traits, we did not observe evidence of an association in the eFHS cohort. Further research is needed to analyze our findings in multiracial cohorts and understand associations between different types of PA—such as aerobic, anaerobic, high interval training—and ECG traits.

## Funding Sources

The Framingham Heart Study acknowledges the support of contracts NO1-HC-25195, HHSN268201500001I, and 75N92019D00031 from the 10.13039/100000050National Heart, Lung and Blood Institute. This study was supported by an award from the 10.13039/100000867Robert Wood Johnson Foundation (number 74624) and a grant from the 10.13039/100000050National Heart, Lung and Blood Institute (R01HL141434); investigator time from the following grants: 2R01 HL092577 (EJB); 1R01AG066010 (EJB), 10.13039/100000968American Heart Association 18SFRN34110082 (EJB) and 18SFRN34150007 (LT), R01HL126911 (DDM), R01HL137734 (DDM), R01HL137794 (DDM), R01HL13660 (DDM), 20SFRN35360180 (HL), and AARG-NTF-20-643020 (HL). Dr Kornej received funding from the Marie Sklodowska-Curie Actions under the European Union’s Horizon 2020 research and innovation programme (Agreement No 838259).

## Disclosures

The Apple Watches were provided to Boston University by Apple Inc at no cost to the study. Apple was not involved in the study design, analysis, interpretation, or reporting of study results.

## Authorship

All authors attest they meet the current ICMJE criteria for authorship.

## Patient Consent

All participants gave written consent.

## Ethics Statement

The study protocol was approved by the Institutional Review Board at the Boston University Medical Center. The research reported in this paper adhered to the Helsinki Declaration as revised in 2013.

## Disclaimer

Given his role as Editor in Chief, David McManus had no involvement in the peer review of this article and has no access to information regarding its peer review. Full responsibility for the editorial process for this article was delegated to David Duncker. Given their role as Associate Editors and Section Editor, Belinda Borrelli, Chunyu Liu, and Honghuang Lin had no involvement in the peer review of this article and have no access to information regarding its peer review.
